# Editorial: Higher Plants, Algae and Cyanobacteria in Space Environments

**DOI:** 10.3389/fpls.2021.629014

**Published:** 2021-02-10

**Authors:** Carmen Arena, Thomas Graham, Valerie Legué, Roberta Paradiso

**Affiliations:** ^1^Department of Biology, University of Naples Federico II, Naples, Italy; ^2^School of Environmental Sciences, University of Guelph, Guelph, ON, Canada; ^3^Université Clermont Auvergne, INRAE, PIAF, Clermont-Ferrand, France; ^4^Department of Agricultural Sciences, University of Naples Federico II, Naples, Italy

**Keywords:** bioregenerative life-support systems, International Space Station, microgravity, photosynthetic organisms, space radiation

Humanity is on the verge of permanently extending its footprint out into the solar system. Before permanence can be achieved, we must first fully understand and harness the power of autotrophic biological systems; systems that, in essence, will be our life-support technology beyond Earth.

It is with this thought in mind that this broad-based collection of research, engineering, and review articles is presented. The overall theme reflects spaceflight and ground-based space analog experiments and engineering concepts that contribute to the development and understanding of bioregenerative life-support systems that will enable long-duration human space exploration and colonization.

The issue is further focused on studies and concepts that hold autotrophic (photosynthetic) organisms at the center of the technological enterprise. These primary producers, including higher plants and photosynthetic algae and cyanobacteria, are the foundation of natural ecosystems on Earth. The energy fixed by these autotrophic organisms is transferred along complex food webs, ultimately sustaining the vast majority of life on Earth. During this process, these organisms also are continually contributing to the recycling or revitalization (from a human perspective) of our air and water through photosynthesis and transpiration. Based on their foundational roles on the Earth, it stands to reason that autotrophs will be equally necessary for human survival on other planetary bodies and human spaceflight environments (e.g., long duration transit missions). Hence, the possibility of growing plants, photosynthetic algae and cyanobacteria in extraterrestrial environments represents an open challenge for scientists.

This volume represents the most recent studies, reviews, and concept developments on the cultivation of autotrophs in spaceflight and spaceflight relevant settings. The articles cover a broad swath of topics that take into account the multiple constraints imposed by the challenges of spaceflight: e.g., severe volume restrictions in habitats, power requirements/limitations, cosmic radiation, altered gravity, altered atmospheric compositions, and their combined effects.

Higher plants, as well as algae and other photosynthetic organisms, are fundamental in these life-support systems as they are the primary food producers, although they also rely on other supporting biological systems. As regards algae, it has been demonstrated that they may grow and photosynthesize in Space, because algae can survive to direct exposure to space environment, however the combined effects of microgravity and radiation on these organisms are still not assessed (Niederwieser et al., 2018).

The selection and/or development of suitable photosynthetic organism as candidates for bioregenerative life-support systems (BLSSs) is currently a priority research area in human space exploration. The eligible traits for the selection of algal strains for long term human spaceflights are the fast growth and elevated photosynthetic rates and the capacity to cope with space factors (Wang et al., 2006). As regards higher plants, the crop varieties need to meet diverse requirements, including but not limited to, compact architecture (volume utilization efficiency), short production cycles, high productivity (e.g., high harvest index), high nutritional quality including nutraceutical/functional food considerations, and tolerance to novel or extreme radiation or gravitational environments.

Studies carried out directly in Space, on space vehicles, or on the International Space Station (ISS), offer a great opportunity for scientists to better understand plant responses to multiple [novel] stressors. Although critical, access to space is limited; as such, ground-based analog studies also play a key role in advancing our understanding of plant space biology.

Ground-based alternatives to spaceflight experiments facilitate early exploratory studies and/or validate spaceflight results where appropriate. Instruments such as clinostats, random-positioning machines (RPM), and magnetic levitation devices have been used to offset the constant gravity vector on plant growth and development. Kiss et al. provide a review of the strengths and weaknesses of these analog systems for the study of plant growth and development. They compare spaceflight and ground experiments and discuss the methods and considerations of these. Keeping these limitations in mind, several ground-based experiments examine the gravity response of root, essential for understanding of the behavior of plants in microgravity environment. A study by de Bang et al. examined autotropism on root development, a process in which the roots straightened after the initial gravistimulus was withdrawn by clinorotation. Combining pharmacological experiments with 2D-clinoratation and actin observation, the authors show the implication of brassinosteroids in root autotropism and suggest that brassinosteroids modulate this process by modifying F-actin organization and dynamics in a manner different from that of the actin-disrupting compound LatB. These studies reinforce previous observations that the acto-myosin system is an important component of plant tropic responses.

In this issue, ground-based experiments review and examine the effects of simulated space radiation on several species (Mousseau and Moller; Desiderio et al.; Zhang et al.; Barker et al.). These studies aim to better understand the molecular mechanisms of plant resistance, in which antioxidant compounds have a key role. Enhanced production of pharmaceutically relevant molecules, including antioxidants as well as other metabolites and recombinant proteins, in response to radiation may represent a new and important frontier for space research. The outcomes of the effects of chronic low-dose rate radiation exposure have also been considered in the light of studies conducted on Earth and in particular in Chernobyl, Fukushima, and other regions of the world with high ambient radiation levels, considering the interactions between radiation and other environmental stressors (e.g., temperature, drought, heavy metals), that may play important roles in determining sensitivity to radiation induced stress (Mousseau and Moller).

Gravity, or gravitropism, is a major terrestrial factor influencing plant development and orientation. On Earth, other factors including light, water, nutrients also influence the direction of plant growth and their respective role may be masked by gravitropism. In this context, the review by Muthert et al. offers a summary of current knowledge on root tropisms to different environmental stimuli. The authors describe the importance of *Arabidopsis thaliana* as a model organism in tropism research. They also highlight the importance of investigating the signaling pathways and their interactions in other species to get a more comprehensive view of tropisms. Thanks to MULTITROP (Multiple-Tropism: interaction of gravity, nutrient and water stimuli for root orientation in microgravity), performed on the ISS, Izzo et al. disentangled hydrotropism from chemotropism for root orientation in the absence of the gravity stimulus. The analysis of root development and orientation of *Daucus carota* seedlings grown in microgravity and at 1 g showed a positive chemotropism of roots toward nutrients in the absence of the gravity stimulus. In the study proposed by Califar et al. and his collaborators, an Advanced Plant Experiments-03- 2 (APEX-03-2) spaceflight study was designed to elucidate the contribution of two skewing- and cell wall- associated genes in Arabidopsis to root behavior and gene expression patterns in spaceflight. Moreover, the authors offer a complete picture of the physiological and metabolic processes appearing to be affected under spaceflight conditions. In their paper, Herranz et al. address the complex interaction between phototropism and gravitropism. The authors attempt to dissect the molecular mechanisms behind this process through an RNA-seq analysis of blue-light irradiated *Arabidopsis* seedlings grown in an onboard centrifuge to generate different gravity levels. The main results demonstrated that genes differentially expressed at low g or lunar g levels are not present in blue-light irradiated plants, suggesting that blue light might reduce gravitational stresses in microgravity.

Always on the subject of studies on microgravity, Monje et al. have performed a hardware validation test to demonstrate that the Advanced Plant Habitat (APH) facility was fully operational and capable of conducting fundamental plant research in microgravity onboard the ISS.

Fundamental plant science advances driven by studying the response of plants to the unique conditions presented by the spaceflight environment have pushed the boundaries of our understanding of plant molecular biology (Herranz et al.; Izzo et al.). Although pure plant science advances are tremendously important, it can be argued that the fundamental objective of studying plants and other autotrophic organisms in spaceflight environments is to support the development of future life-support systems that are based on these autotrophic organisms and the biological communities that support them (Graham and Bamsey, 2016). Given this fundamental objective, it is no surprise that much of the research and commentary presented in this collection is focussed on the production systems and autotrophic species that will 1 day support human life beyond low earth orbit, while also improving agricultural production here on Earth.

Bioregenerative life-support is simple in principle but complex in its execution. Many questions remain with respect to how candidate crops will respond to, or can be adapted to thrive in, the spaceflight environment. Beyond the plants themselves, there is still a good deal of work to be done to design and refine the systems that will provide the appropriate environment(s) to sustain the biology that humans will rely on to provide the key elements of life-support. Research presented in this collection addresses important issues relating to plant adaptability to spaceflight and related ground conditions, the choice of the most suitable cultivation system(s), and the provision of optimal environmental parameters for specific crop types. Studies include consideration of plant genotypes, overall growth facility designs, sub-system development and characterization (e.g., lighting; air handling), including hydroponic and lighting systems, and growth substrates. Innovative research is presented detailing the leading edge of facility design and operation, such as the EDEN ISS growth chamber that incorporates a plant health monitoring system to provide remote support for plant status assessment and early detection of plant stress or disease (Battistuzzi et al.; Paradiso et al.; Peiro et al.; Zeidler et al.; Rouphael et al.; Zabel et al.). This approach allows new discoveries to be achieved in facilities conceived for, and operated as, analogs to space platforms such as those that 1 day be established on the moon and Mars. In these facilities, higher plants play key roles, by regenerating air through photosynthetic CO_2_ absorption and O_2_ emission, processing and recovering water through transpiration and recycling organic waste products through mineral nutrition. However, in order to optimize their performance, the best genotypes and the optimal growing conditions need to be established. Accordingly, in candidate crops such as potato, ground experiments are presented that evaluate the response of different cultivars to different environment conditions (e.g., light intensity and duration, CO_2_ air concentration; Wheeler et al.). It is critical to understand the specific environmental needs of each crop, as these parameters directly impact the fundamental processes, such as photosynthesis, that sustain plant growth and thereby human life. In parallel, cultivation systems need to be evaluated for their ability to grow safe fresh food to supplement packaged food for astronauts (Khodadad et al.). This is an important consideration for the worlds space agencies, as the spaceflight environment is known to alter plant and algae physiology and composition (Häder; Califar et al.; Izzo et al.; de Bang et al.; Herranz et al.; Stewart et al.). To study the effects of space conditions on microbiological and nutritional aspects, several model crops are grown in Veggie plant growth chamber on the International Space Station (ISS) and compared with ground grown plants (Zhang et al.; Khodadad et al.). Further, space and ground studies by Khodadad et al. and Zeidler et al., respectively, suggest that food production in space is achievable within the confines of food safety standards (Khodadad et al.; Zeidler et al.).

This Research Topic was conceived with the intent to present research, methods, and reviews that contribute to a meaningful risk evaluation for biological systems exposed to extraterrestrial environments. Although the focus of the presented research was on autotrophs, it is the role these autotrophs play in human survival in space that was the real focus. As humanity steps beyond Earth's protective influence to expand our footprint into the solar system, we will need the knowledge and ideas developed by research scientists and engineers, such as those highlighted in this Research Topic, if we are to thrive as a spacefaring species.

## Author Contributions

CA, VL, TG, and RP: conceptualization. CA, VL, TG, and RP: writing original draft. All authors: writing—review and editing.

## Conflict of Interest

The authors declare that the research was conducted in the absence of any commercial or financial relationships that could be construed as a potential conflict of interest.
